# Draft Genome Sequences of Macrococcus caseolyticus, Macrococcus canis, Macrococcus bohemicus, and Macrococcus goetzii

**DOI:** 10.1128/MRA.00343-19

**Published:** 2019-05-09

**Authors:** Shahneela Mazhar, Eric Altermann, Colin Hill, Olivia McAuliffe

**Affiliations:** a Teagasc Food Research Centre, Moorepark, Fermoy, Cork, Ireland; b School of Microbiology, University College Cork, Cork, Ireland; c Animal Science Group, AgResearch, Palmerston North, New Zealand; d APC Microbiome Institute, Cork, Ireland; e Riddet Institute, Massey University, Palmerston North, New Zealand; Broad Institute of MIT and Harvard University

## Abstract

Here, we present the draft genome sequences of 14 strains of 4 species of the genus *Macrococcus*. These strains were isolated from bovine milk and tongue samples obtained during a screening program.

## ANNOUNCEMENT

Fourteen strains belonging to four members of the *Macrococcus* genus, namely, 3 Macrococcus caseolyticus strains (DPC 6291, DPC 7170, and DPC 7171), 7 Macrococcus canis strains (DPC 7158, DPC 7160, DPC 7162, DPC 7163, DPC 7165, DPC 7168, and DPC 7169), 3 *Macrococcus goetzii* strains (DPC 7159, DPC 7164, and DPC 7166), and 1 *Macrococcus bohemicus* strain (DPC 7215), were isolated from bovine milk and tongue by utilizing a *ctaC* PCR, as described previously (1). Recently emerging information regarding multidrug resistance and putative virulence genes present in species belonging to this genus prompted us to perform whole-genome sequencing (WGS) to investigate the presence of such genes in these *Macrococcus* strains (2–4).

The genomic DNA was isolated from overnight cultures grown at 37°C in tryptic soy broth (TSB; Becton, Dickinson and Company, Berkshire, England) using the UltraClean microbial DNA isolation kit (Mo Bio Laboratories, Cambridge, UK) as per the included protocol. Genomic libraries were prepared with a Nextera XT DNA library preparation kit (Illumina, Inc., San Diego, CA, USA). The 2 × 250-bp paired read sequencing was performed on an Illumina HiSeq 2500 platform (MicrobesNG, University of Birmingham, UK). Reads were adapter trimmed using Trimmomatic version 0.30, with a sliding window quality cutoff of Q15 (5). *De novo* assembly was performed on each sample using SPAdes version 3.7 with the program’s default parameters (6). Detection of acquired antimicrobial resistance genes in the assembled genomes was analyzed using ResFinder version 3.4 and Resistance Gene Identifier (RGI) version 4.2.2 to search against the Comprehensive Antibiotic Resistance Database (CARD). Virulence genes were identified using VirulenceFinder version 2.0, PathogenFinder version 1.1, and the Virulence Factors Database (VFDB) (7–10). The genome sequences were annotated using the NCBI Prokaryotic Genome Annotation Pipeline (PGAP) (11). The final draft genomes were estimated using CheckM (12) to be ≥96% complete with <2.5% contamination.

All sequenced genomes illustrated the presence of putative virulence factors, namely, hemolysin III (*hlyIII*), aureolysin (*aur*), and capsule (*cap*) genes. An RGI search of the homology models in CARD identified a total of 86 different antibiotic resistance genes, most of which are predicted to confer resistance to fluoroquinolone (*n* = 19), macrolides (*n* = 26), and tetracycline (*n* = 24). The sequencing and assembly statistics of the draft genome sequences of the above-mentioned *Macrococcus* strains are shown in Table 1. The sequencing data contribute to the pool of available *Macrococcus* genomes and enable further generation of information regarding the presence of antibiotic resistance determinants and other virulence factors present in *Macrococcus* species.

**TABLE 1:** Genome characteristics of the *Macrococcus* strains used in this study

Organism	SRA accession no.	GenBank accession no.	Draft genome size (bp)	G+C content (%)	No. of contigs	Coverage (×)	*N*_50_ (bp)
*M. caseolyticus* DPC 6291	SRR8868656	SDQM00000000	2,171,480	36.68	74	70	229,924
*M. canis* DPC 7158	SRR8868660	SDQI00000000	2,179,466	36.75	69	197	578,934
*M. goetzii* DPC 7159	SRR8868665	SDGN00000000	2,530,812	34.06	93	184	275,573
*M. canis* DPC 7160	SRR8868666	SDQF00000000	2,148,516	36.58	37	136	413,516
*M. canis* DPC 7162	SRR8868667	SDQG00000000	2,139,904	36.62	44	107	353,259
*M. canis* DPC 7163	SRR8868668	SDQH00000000	2,167,812	36.63	79	147	417,178
*M. goetzii* DPC 7164	SRR8868659	SDGO00000000	2,563,253	34.07	61	137	458,326
*M. canis* DPC 7165	SRR8868658	SDGP00000000	2,165,327	36.68	72	158	1,280,134
*M. goetzii* DPC 7166	SRR8868662	SDGQ00000000	2,591,067	34.16	95	202	466,093
*M. canis* DPC 7168	SRR8868661	SDGR00000000	2,134,151	36.68	41	95	397,880
*M. canis* DPC 7169	SRR8868664	SDGS00000000	2,160,199	36.56	89	264	1,113,524
*M. caseolyticus* DPC 7170	SRR8868655	SDQK00000000	2,106,646	36.77	67	48	147,285
*M. caseolyticus* DPC 7171	SRR8868657	SDQJ00000000	2,110,528	36.77	99	231	108,839
*M. bohemicus* DPC 7215	SRR8868663	SELR00000000	2,555,877	33.98	55	160	234,144

### Data availability.

The draft WGS data were deposited into NCBI GenBank and the Sequence Read Archive (SRA) under the BioProject no. PRJNA515496. The accession numbers are listed in Table 1.
